# Discrepant Activation Pattern of Inflammation and Pyroptosis Induced in Dermal Fibroblasts in Response to Dengue Virus Serotypes 1 and 2 and Nonstructural Protein 1

**DOI:** 10.1128/spectrum.03586-22

**Published:** 2023-01-11

**Authors:** Kai-Che Wei, Wan-Ju Wei, Ching-Len Liao, Tsung-Hsien Chang

**Affiliations:** a Department of Dermatology, Kaohsiung Veterans General Hospital, Kaohsiung, Taiwan; b School of Medicine, National Yang Ming Chiao Tung University, Taipei, Taiwan; c National Institute of Infectious Diseases and Vaccinology, National Health Research Institutes, Miaoli, Taiwan; d Department and Graduate Institute of Microbiology and Immunology, National Defense Medical Center, Taipei, Taiwan; Center for Research and Advanced Studies

**Keywords:** dengue virus, NS1, pyroptosis, caspase-1, IL-1β, dermal fibroblasts

## Abstract

Four serotypes of dengue virus (DENV-1 to DENV-4) cause mild to severe disease in humans through infected mosquito bites. Dermal fibroblasts were found to be susceptible to DENV, and this may play a critical role in establishing the initial infection stage. However, the cellular response induced by the different DENV serotypes in dermal fibroblasts during the early stage of infection remains unclear. To determine this, normal human dermal fibroblast WS1 cells were infected with DENV-1 or DENV-2. Compared with the response elicited by DENV-1 infection, DENV-2 induced a stronger innate inflammatory response and cell death in the WS1 cells. However, DENV-1 activated a higher level of pyroptosis signaling than did DENV-2, which was associated with higher virion production. Caspase-1 inhibitor Ac-YVAD-cmk and imipramine, an antidepressant drug, reduced DENV-mediated caspase-1 and interleukin 1β (IL-β) cleavage in the pyroptosis pathway. Ac-YVAD-cmk and imipramine downregulated DENV virion production in WS1 cells. Furthermore, DENV-1 and DENV-2 NS1 proteins promoted diverse activation levels of cell death, inflammatory response, and activation of caspase-1 and IL-β in dermal fibroblasts at different time points. Collectively, these data suggest that DENV-1, DENV-2, and their nonstructural protein 1 (NS1) induce discrepant activation patterns of inflammation and pyroptosis in dermal fibroblasts. The pyroptosis caused by virus and NS1 may facilitate DENV replication in dermal fibroblasts.

**IMPORTANCE** Skin fibroblasts are the primary cells of DENV infection through mosquito bites. Establishing a successful infection in dermal fibroblasts might be critical for dengue disease. However, the cellular response induced by DENV in dermal fibroblasts remains unclear. In this *in vitro* study, we found that DENV-2 and DENV-1 showed different time course patterns of virus replication and inflammation in dermal fibroblasts. We demonstrated that DENV-1 and DNEV-2 and their viral protein NS1 activate the cellular pyroptosis response to regulate virus replication in dermal fibroblasts. This finding suggests that pyroptosis activation in the DENV primary inoculation site plays a role in the establishment of a DENV infection.

## INTRODUCTION

Dengue is a mosquito-borne viral infection prevalent in tropical and subtropical regions of the world. Dengue is caused by the dengue virus (DENV), a member of *Flaviviridae* and classified into four serotypes, DENV-1 to DENV-4. The DENV virion possesses an 11-kb single-stranded genomic RNA, which encodes a polyprotein. Viral or host proteases further cleave DENV polyproteins into three structural proteins (C, PrM/M, and E) and seven nonstructural proteins (NS1, NS2A, NS2B, NS3, NS4A, NS4B, and NS5). These proteins are responsible for the formation of DENV virus particles (1). Symptoms of dengue range from subclinical or mild to fatal complications. Treating severe dengue is challenging, as antiviral medicines are currently unavailable for dengue other than for supportive therapy. The global incidence of dengue has increased sharply in recent decades. It is estimated that up to 400 million people are infected with DENV, with 40,000 deaths, each year. In some Asian and Latin American countries, severe dengue is often the leading cause of serious illness and death (1–3). Meta-analysis revealed that DENV-2 and DENV-4 caused more cases of dengue hemorrhagic fever, dengue shock syndrome, and severe dengue disease than did DENV-1 (4, 5). However, the reason for this discrepancy is unclear.

Cell death is a part of the host defense machinery that limits replication of the invasive viruses within cells; however, various viruses have been found to promote their replication using the cell death pathway (6). In general, the mechanisms causing cell death can be classified into apoptosis, necrosis, autophagy, and pyroptosis (7). Apoptosis and pyroptosis occur during DENV replication in host target cells (8). Several studies have shown that DENV infection causes apoptosis in different cell types, such as hepatocytes, neuronal cells, endothelial cells, and monocytes (9); DENV-induced mitochondrial and endoplasmic reticulum stress-mediated apoptosis has also been reported (10, 11). DENV-1 to DENV-4 M protein, DENV-1 and DENV-2 C protein, DENV-2 E protein domain III, NS3, or NS2B3, and subgenomic RNA have been identified as proapoptosis factors (9).

Inflammation is a cellular response as a part of innate immunity against viral infections in the body; however, excessive inflammation leads to tissue damage and exacerbates the disease. In contrast to apoptosis, infection-induced pyroptosis is a type of lytic cell death that is accompanied by an inflammatory response. Pyroptosis is characterized by caspase-1-dependent formation of plasma membrane gasdermin D pores, which cause cellular swelling, lysis, and release of inflammatory cytokines, such as interleukin 1β (IL-1β) and IL-18 (12). DENV-induced pyroptosis has been observed in macrophages and monocytes. In human macrophages, DENV-2 induces high levels of IL-1β and IL-18, causes pyroptosis, and upregulates pro-IL-1β, pro-IL-18, and NLRP3, which is associated with caspase-1 activation through myeloid Syk-coupled C-type lectin 5A (13). In human monocytes, DENV-2-triggered caspase-1 activation and IL-1β production have been observed (14), indicating the immunopathogenesis of DENV infections. Nevertheless, it is unclear whether DENV induces pyroptosis in cell types other than macrophages and monocytes.

DENV is transmitted between persons through the bite of infected female Aedes aegypti. Thus, the skin is the initial site of DENV inoculation, making dermal and epidermal cells potentially susceptible to infection. The skin is also the primary site of antiviral immune response initiated after DENV infection (15). DENV-2 infection of the epidermal keratinocytes and dermal fibroblasts results in the activation of toll-like receptor 3 (TLR3)–retinoic acid-inducible gene I (RIG-I) axis-mediated type I interferon (IFN) innate immune response (16, 17). The cell-cell interaction between DENV-infected keratinocytes and recruited virus-permissive myeloid cells, such as Langerhans cells, macrophages, and dermal dendritic cells, may enhance the spread of DENV in human skin. Keratinocyte-expressed IL-1β plays a major role in this response (18). In addition, the interplay of DENV-2-infected dermal fibroblasts with dendritic cells promotes a type I IFN-mediated antiviral response and stimulates T cell proliferation as a part of the adaptive immune response in infected skin (19). These studies suggest that the cross talk between diverse cell types in the microenvironment of DENV-inoculated skin initiates the primary antiviral response, which may regulate DENV dissemination. However, the underlying mechanisms of the cellular response in each specific dermal cell type remain to be investigated.

DENV-induced cell death has not been clearly evaluated in human skin fibroblasts, although host DNA fragmentation has been confirmed using the terminal deoxynucleotidyltransferase-mediated dUTP-biotin nick end labeling assay (16). Since DENV induces the activation of the inflammatory antiviral response in dermal fibroblasts, we hypothesized that pyroptosis-associated inflammation may occur in DENV-infected dermal fibroblasts. DENV NS1 is a hexameric lipoprotein that circulates in high concentrations in patient sera and is an early diagnostic marker for dengue infection. NS1 is a pathogenic factor of DENV that facilitates viral replication and causes hemorrhage and vascular leakage (20, 21). We hypothesized that DENV NS1 is involved in virus-mediated pyroptosis pathway activation. To evaluate our hypothesis, we used WS1, a normal human dermal fibroblast cell line, as the infection cell model for DENV-1 and DENV-2 to demonstrate the activation of the pyroptosis pathway and to determine the effect of the DENV virotoxin NS1 on the cell death pathway.

## RESULTS

### DENV infected dermal fibroblasts and caused cell damage.

**FIG 1:**
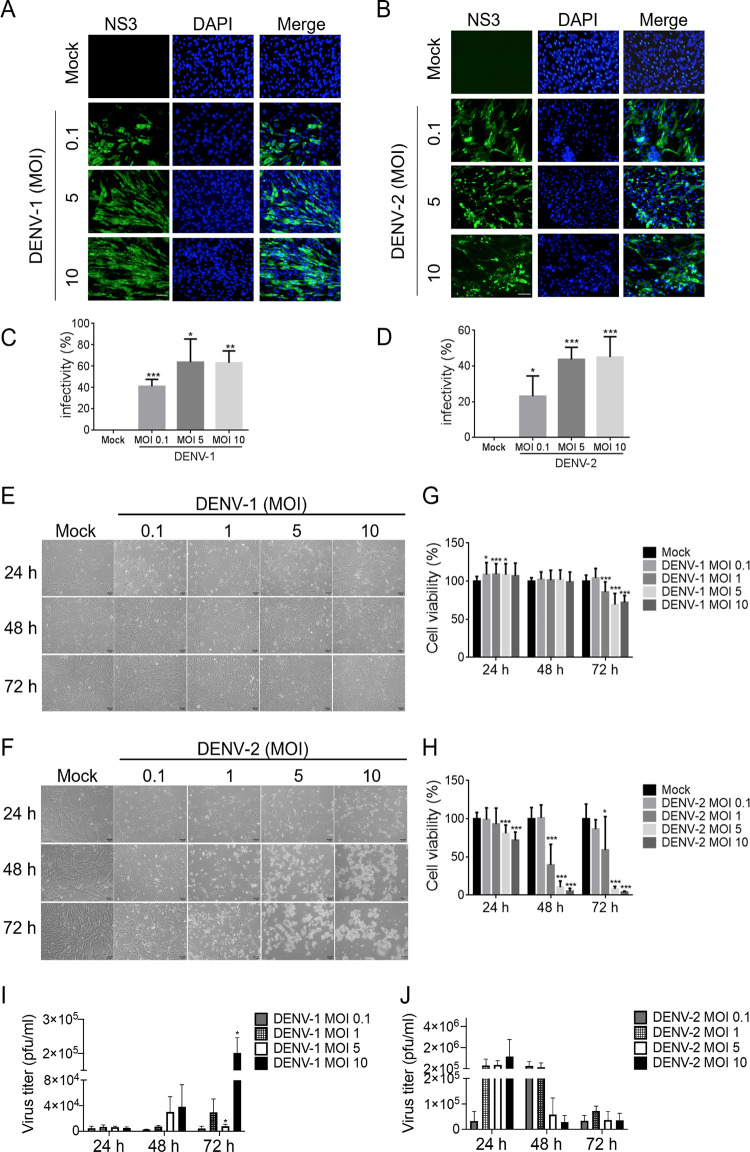
DENV causes cell damage in dermal fibroblasts. (A and B) WS1 cells (5 × 10^4^) were either uninfected (mock) or infected with DENV-1 or DENV-2 at an MOI of 0.1, 5, or 10 for 48 h and then subjected to immunofluorescence assay with anti-NS3 antibody followed by Alexa Fluor 488-conjugated anti-mouse IgG. Nuclei were stained with DAPI. The captured images of NS3 staining (green) and DAPI (blue) by fluorescence microscopy were merged. Scale bar, 1 μm. (C and D) The infectivity of DENV-1 and DENV-2, determined using immunofluorescence assay, was calculated. (E and F) Phase-contrast images showing the cell morphology of WS1 cells with DENV-2 infection (MOI, 0.1, 1, 5, and 10) at 24, 48, and 72 h. Magnification, ×100. Scale bar, 1 μm. (G and H) Luminescence cell viability assay was conducted in WS1 cells with different DENV-1 and DENV-2 viral loads of at each induction time point. Statistical analysis used Student's *t* test. *, *P* < 0.05; **, *P* < 0.01; ***, *P* < 0.005 compared with mock control. (I and J) The culture medium with WS1 cells infected with DENV-1 or DENV-2 (MOI, 0.1, 1, 5, or 10), which were harvested at 24, 48, and 72 h. The titers of DENV virion production were determined using a plaque assay. Statistical analysis used Student's *t* test. *, *P* < 0.05; **, *P* < 0.01; ***, *P* < 0.005 compared with the group at 24 h postinfection.

Skin is the initial inoculation site for DENV infection, and to understand whether dermal fibroblasts are susceptible, an immunofluorescence assay was conducted in WS1 cells (normal human dermal fibroblasts) infected with DENV-1 or DENV-2 at a multiplicity of infection (MOI) of 0.1, 5, or 10 for 48 h. NS3 staining was used to indicate DENV-1- and DENV-2 =-infected cells (Fig. 1A and B), and a higher infectivity was measured with a higher MOI infection (Fig. 1C and D). The viral infection-induced cytopathic effect (CPE) was not visible in DENV-1-infected WS1 cells (Fig. 1E), but severe CPE was observed in dermal fibroblasts infection with DENV-2 (Fig. 1F). We further examined the viability of WS1 cells following DENV infection at different viral loads and infection periods. High-MOI infection of DENV-1 reduced WS1 cell proliferation at the late phase of the infection period (72 h) by approximately 25% (Fig. 1G). However, starting at 24 h postinfection, DENV-2 infection significantly impaired dermal fibroblast proliferation, which worsened at 48 and 72 h compared with that in the mock control; less than 10% of cell viability was detected in high-viral-load infection (MOI of 5 or 10) at 48 and 72 h (Fig. 1H). Since different levels of CPE were observed between DENV-1 and DENV-2 infection, the titers of virion-producing WS1 cells were analyzed. As expected, DENV-1 titer increased in a viral load-dependent manner during the infection period (Fig. 1I), whereas a decreased viral titer was detected in dermal fibroblasts with high-MOI infection of DENV-2 at 48 and 72 h (Fig. 1J). These data suggested that DENV targets dermal fibroblasts and causes cell death, which might modulate viral replication.

### DENV induced an inflammatory antiviral response in dermal fibroblasts.

**FIG 2:**
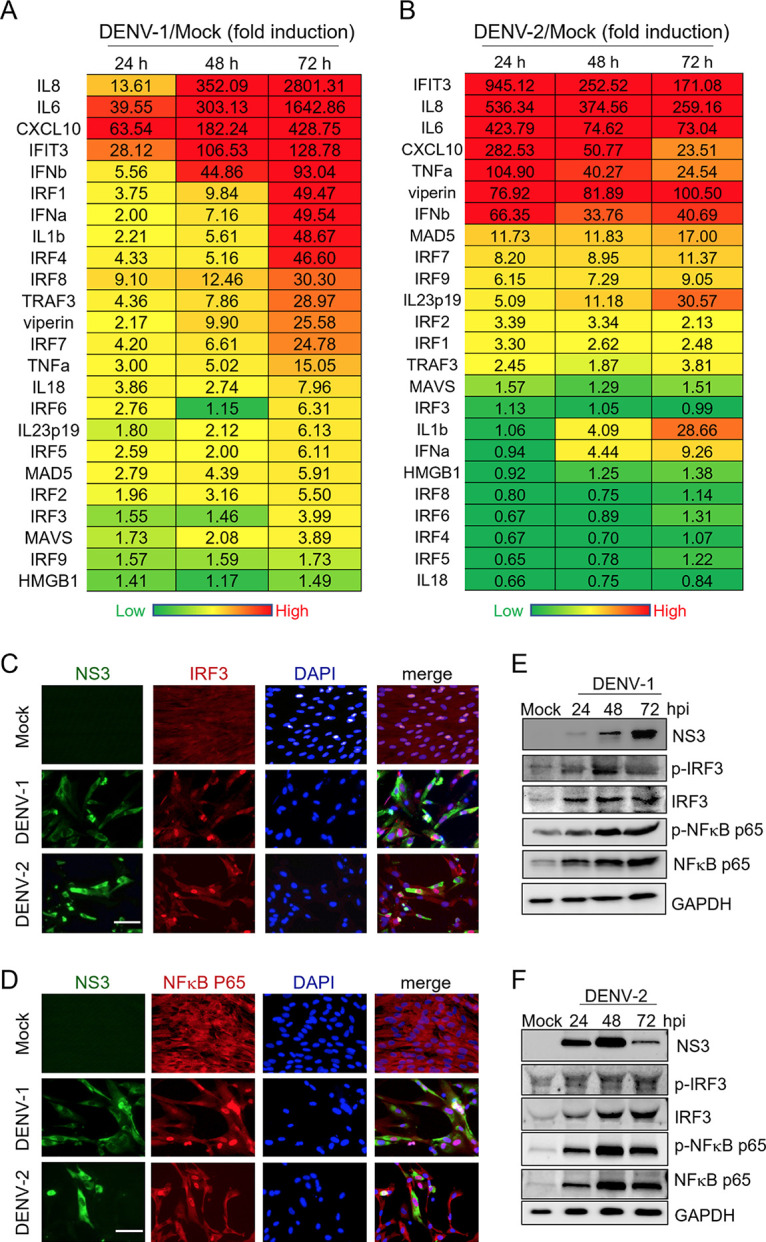
DENV-induced inflammatory antiviral responses in dermal fibroblasts. (A and B) A total of 1 × 10^6^ WS1 cells were uninfected (mock) or infected with DENV-1 or DENV-2 at MOI of 1 for 24, 48, and 72 h. The level of mRNA expression of antiviral inflammation genes, cell response genes, signaling pathway genes, and IRF family genes were determined using qRT-PCR. The gene expression was normalized to that of GAPDH. The mean fold induction (infection/mock, *n* = 3 each) of each gene was visualized and colored. (C and D) Immunofluorescence assay was conducted in WS1 cells (5 × 10^4^) to determine the IRF3 and NF-κB p65 cellular location for DENV-1 or DENV-2 infection at an MOI of 5 for 72 h. The cellular locations of IRF3 and NF-κB p65 (red fluorescence) and DENV-infected cells (detection of NS3; green fluorescence) were visualized. Nuclei were stained with DAPI (blue). Merged images are shown. Scale bar, 1 μm. (E and F) Immunoblotting assay was conducted in WS1 cells with DENV-1 or DENV-2 infection at an MOI of 1 at 24, 48, and 72 h. The protein expression of DENV-NS3, phospho-IRF3, total IRF3, phospho-NF-κB p65, total NF-κB p65, and GAPDH were determined in a plaque assay.

Virus-mediated cell death is frequently accompanied by inflammation. Hence, we evaluated if inflammatory antiviral response flared in DENV-infected dermal fibroblasts. Quantitative PCR (qPCR) analysis showed that DENV-1- or DENV-2-infected WS1 cells expressed tremendously high levels of the genes related to inflammatory cytokines and IFN, such as those for IL-8, IL-6, tumor necrosis factor alpha (TNF-α), CXCL-10, IL-1β, IFN-α, IFN-β, IFIT3, and viperin, and those for interferon regulatory factors (IRFs). In DENV-1-infected WS1 cells, the responses gradually increased 24 to 72 h postinfection (Fig. 2A). DENV-2 induced the highest level of inflammatory antiviral response at 24 h postinfection, and the level decreased at the later periods (48 and 72 h) (Fig. 2B). This pattern of dynamic change in inflammatory response correlated with the difference between DENV-1- and DENV-2-induced dermal fibroblast death, suggesting an association between cell death and inflammation.

To further confirm the induction of an inflammatory antiviral response by DENV in dermal fibroblasts, the activation levels of the transcription factors IRF-3 and NF-κB p65 were evaluated by determining nuclear translocation and protein phosphorylation. An immunofluorescence assay showed that DENV-1- and DENV-2-infected cells presented the nuclear translocation of IRF-3 and NF-κB p65 in WS1 cells (Fig. 2C and D). Immunoblotting also detected phosphorylated IRF-3 and NF-κB p65 in DENV-1- and DENV-2-infected cells (Fig. 2E and F).

### DENV activated caspase-1 and IL-1β in dermal fibroblasts.

**FIG 3:**
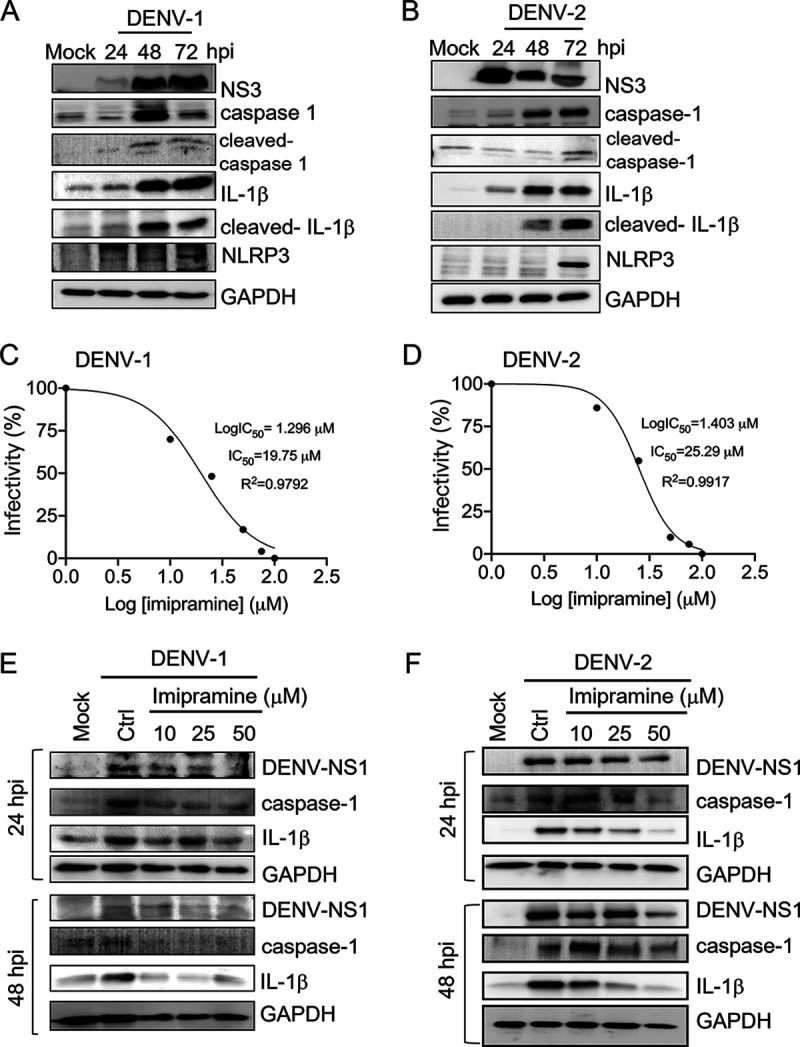
DENV-1 and DENV-2 activate caspase-1 and IL-1β. (A and B) Cell lysates were harvested from WS1 cells with DENV-1 and DENV-2 infection at an MOI of 1 at 24, 48, and 72 h. The immunoblotting analysis was conducted with antibody against DENV-NS3, caspase-1, cleaved caspase-1, IL-1β, cleaved IL-1β, NLRP3, and internal loading control (GAPDH). (C and D) Imipramine inhibition of DENV-1 and DENV-2 infection. WS1 cells were pretreated with vehicle or various doses of imipramine as indicated for 24 h before exposure to DENV-1 and DENV-2 at an MOI of 1. After 2 h of adsorption and 24 h of incubation, an in-cell Western assay was performed with anti-NS3 to detect infected cells. The integrated fluorescence intensity was quantified by the Odyssey CLx near-infrared fluorescence imaging system. The half-maximal inhibitory concentrations (IC_50_) of dose-response curves indicated virus inhibition. Nonlinear regression analysis was used to calculate the IC_50_s by plotting log inhibitor versus normalized response (variable slope). (E and F) The imipramine-treated (10, 25, and 50 μM) WS1 cells were infected with DENV-1 and DENV-2 (MOI of 1) for 24 and 48 h. The cell lysates were harvested for performing an immunoblotting assay of NS1, caspase-1, IL-1β, and the internal loading control, GAPDH.

Pyroptosis is a type of cell death accompanied by inflammation that has not been fully evaluated in DENV infection. Our data suggested a correlation between DENV-mediated cell death and the inflammatory antiviral response. We therefore speculated that DENV-2 infection-induced pyroptosis might be triggered in dermal fibroblasts. Immunoblotting showed that DENV-1 and DENV-2 increased full-length caspase-1 and IL-1β expression and cleavage, the signatures of pyroptosis; DENV-1 and DENV-2 also induced the expression of inflammasome NLRP3 in the pyroptosis signaling pathway (Fig. 3A and B).

The DENV-induced pyroptosis was determined in another dermal cell type, keratinocyte HaCaT cells. Both DENV-1 and DENV-2 infections caused obvious CPE in HaCaT cells (see Fig. S1A in the supplemental material). Compared to DENV-1, DENV-2 caused a higher level of cytotoxicity in HaCaT cells (Fig. S1B). The DENV-1 virion production was slightly downregulated during the infection period, and the DENV-2 virion production decreased dramatically at 72 h postinfection (Fig. S1C and D). These decreased levels of DENV-1 and DENV-2 virion production might have been due to the low level of cell viability in DENV-infected HaCaT cells. Because DENV-1 caused a low level of CPE in WS-1, DENV-1 virion production was increased during the infection period (Fig. 1E and G). These results also suggested that keratinocytes were more susceptible to DENV infection than dermal fibroblasts. In addition, DENV-1 and DENV-2 also induced IL1β and IL-18 mRNA expression. The protein expression of IL-1β, cleaved IL-1β, caspase-1, cleaved caspase-1, and NLRP3 were detected in HaCaT cells with DENV infection (Fig. S1G and H). These data demonstrated DENV-induced pyroptosis keratinocytes.

Imipramine, an FDA-approved antidepressant drug, has been reported to be able to inhibit flavivirus replication by affecting both the fusion and replication steps of the viral life cycle in primary human skin fibroblasts (22). We determined the cytotoxicity of imipramine in WS1 cells (Fig. S2A), and low-cytotoxicity dosages were used. Imipramine exerted the same inhibitory effect against DENV-1 and DENV-2 in WS1 (50% inhibitory concentrations [IC_50_s] of 19.75 μM and 25.29 μM, respectively) (Fig. 3C and D). Due to the DENV inhibition activity of imipramine, DENV-1-induced caspase-1 and IL-1β expression levels were downregulated by imipramine pretreatment (Fig. 3E and F). This result indicated that DENV-induced caspase-1 and IL-1β expression in WS1 cells depends on viral replication.

### A caspase-1 inhibitor downregulated DENV-2 replication and DENV-2-mediated cell death.

**FIG 4:**
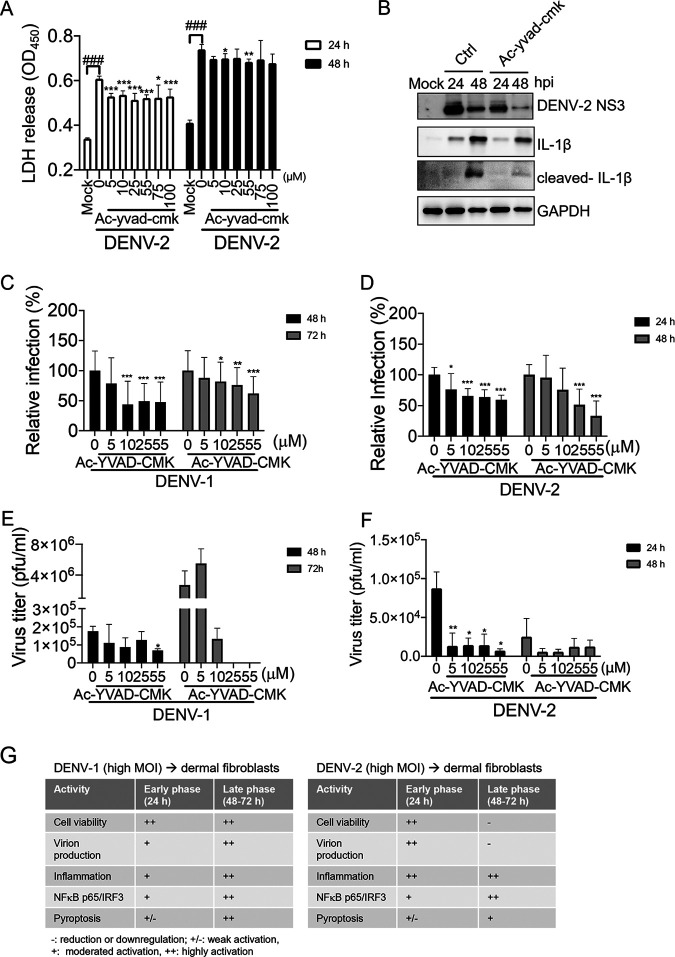
A caspase-1 inhibitor downregulated DENV-2-mediated cell death and DENV-2 replication. (A) WS1 cells were treated with caspase-1 inhibitor Ac-YVAD-cmk (5 to 100 μM) or dimethyl sulfoxide (DMSO) control for 24 h and then mock-treated or infected with DENV-2 (MOI of 1). After infection for 24 and 48 h, the culture medium was subjected to an LDH release assay. Statistical analysis used Student's *t* test. Comparisons with mock infection: ^###^, *P* < 0.005. Comparisons with DMSO control; *, *P* < 0.05; **, *P* < 0.01; ***, *P* < 0.005. (B) WS1 cells were treated with caspase-1 inhibitor Ac-YVAD-cmk 55 μM) or DMSO control for 24 h and then mock-treated or infected with DENV-2 (MOI of 1) for 24 and 48 h. The indicated protein expression was detected in an immunoblotting assay. (C and D) WS1 cells were pretreated with vehicle or various doses of Ac-YVAD-cmk (5 to 55 μM) as indicated for 24 h before exposure to DENV-1 and DENV-2 at an MOI of 1. After 24 and 48 h of infection, an in-cell Western assay with anti-NS3 was performed to detect infected cells. Statistical analysis used Student's *t* test. *, *P* < 0.05; ***, *P* < 0.005 (compared with the untreated control group). (E and F) Ac-YVAD-cmk-pretreated WS1 cells were infected with DENV-1 and DENV-2. The virus yield was determined in a focus-forming assay (FFA) at 24 and 48 h. *, *P* < 0.05; **, *P* < 0.001 (Student’s *t* test, comparing with the untreated control group. (G) Summary of DENV-1 and DENV-2 infection-induced cellular responses in dermal fibroblasts.

We used the caspase-1 inhibitor Ac-YVAD-cmk (*N*-acetyl-tyrosyl-valyl-alanyl-aspartyl chloromethyl ketone) to understand the role of caspase-1 activity in DENV-2 infection of WS1 cells. Dosages of Ac-YVAD-cmk without obvious cytotoxicity concern were applied in this assay (Fig. S2). The lactate dehydrogenase (LDH) cytotoxicity assay showed that DENV-1- and DENV-2-induced cell damage was downregulated by pretreatment with caspase-1 inhibitor (Fig. 4A). Immunoblotting results demonstrated that the caspase-1 inhibitor efficiently reduced downstream molecular IL-1β activation; however, we noticed that the level of DENV NS3 expression was downregulated by Ac-YVAD-cmk (Fig. 4B). We confirmed this observation using immunofluorescence staining of NS3 in WS1 cells after caspase-1 inhibitor pretreatment, and we found that DENV-1 and DENV-2 infectivity was inhibited by Ac-YVAD-cmk in a dose-dependent manner (Fig. 4C and D). Furthermore, DENV-1 and DENV-2 virion production was inhibited by a caspase-1 inhibitor (Fig. 4E and F). These results revealed the potential anti-DENV activity of caspase-1 inhibitor, suggesting that pyroptosis activity might be involved in the DENV-1 and DENV-2 replication process.

The cellular response of dermal fibroblasts to DENV-1 or DENV-2 infection at a high MOI was summarized for the early phase (24 h) and late phase (48 and 72 h) postinfection. DENV-1 replication increased during the infection period without causing severe cell damage. The inflammation level and IRF-3, NF-κB p65, and pyroptosis activities increased in proportion to the virus titers. DENV-2 caused cell death as early as 24 h postinfection, and severe cell damage was detected in the late phase of infection. Cell damage impaired DENV-2 virion production in the late phase. DENV-2 also induced strong inflammation in the early phase and sustained inflammation in the late phase of infection. IRF3/NF-κB p65 and pyroptosis activation were also detected (Fig. 4G). We hypothesized that DENV-1 induces cellular pyroptosis activity without severe inflammation and that cell damage might benefit its replication in dermal fibroblasts. In contrast, although pyroptosis might be involved in DENV-2 replication, the strong inflammation that accompanied DENV-2-induced cell damage would have a negative effect on DENV-2 replication.

### DENV NS1 induced inflammatory cytokine expression in dermal fibroblasts.

**FIG 5:**
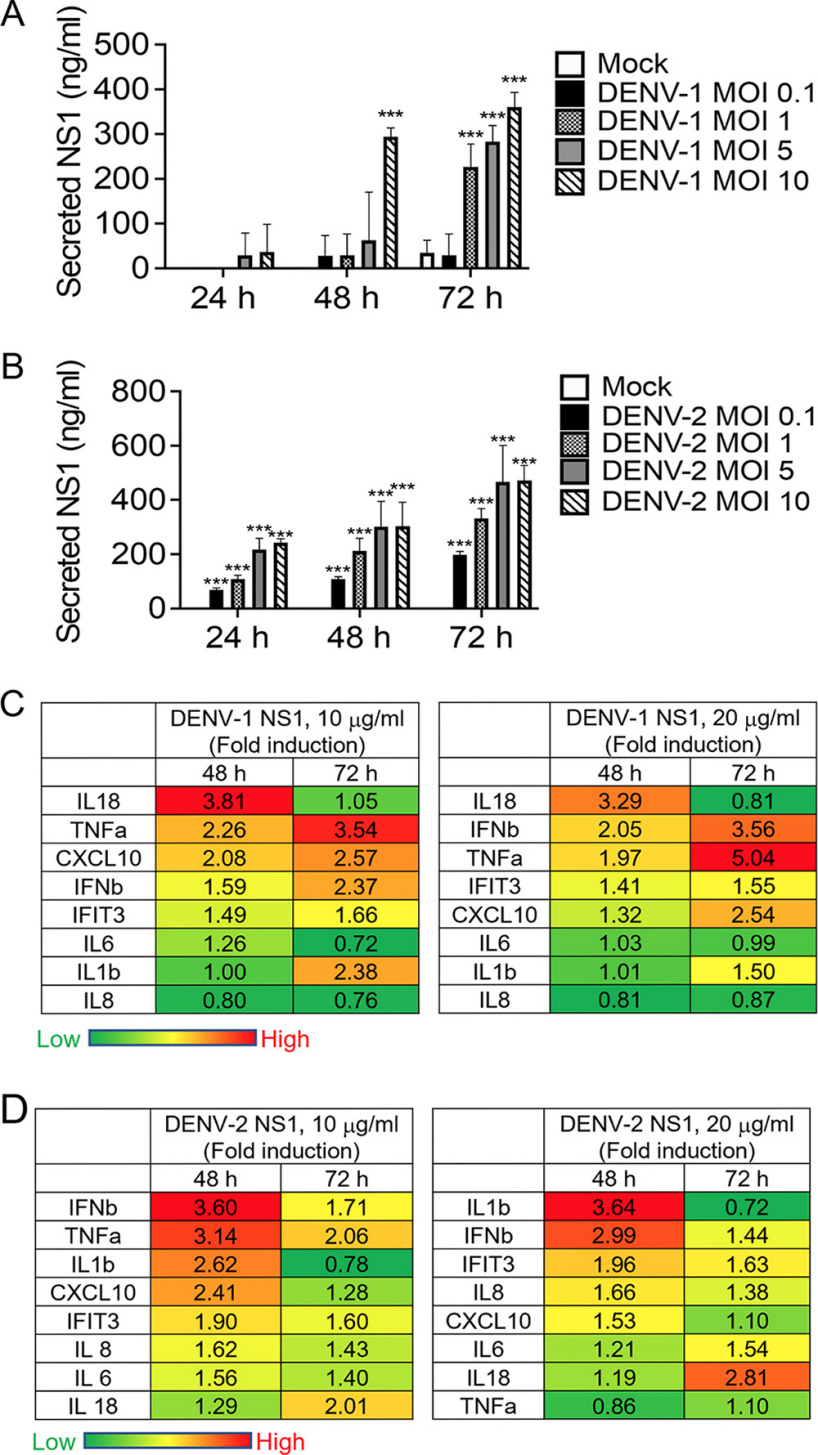
DENV NS1 induced inflammatory cytokine expression in dermal fibroblasts. (A and B) In culture media with DENV-1- or DENV-2-infected (MOI of 0.1, 1, 5, and 10) WS1 cells, which were harvested at 24, 48, and 72 h, secreted NS1 was determined using a dengue NS1 ELISA kit. Statistical analysis was with Student's *t* test. *, *P* < 0.05; **, *P* < 0.01; ***, *P* < 0.005 compared with the mock groups. (C and D) WS1 cells (2 × 10^5^) were treated with recombinant DENV-1 and DENV-2 NS1 (10 and 20 μg/mL) for 48 and 72 h. The mRNA expression levels of *IL-1β*, *IL-6*, *IL-18*, *CXCL10*, *IL-8*, *TNF-α*, *CXCL10*, *IFN-β*, and *IFIT3* were determined using RT-PCR. The results were normalized with the internal control gene, *GAPDH*. The fold induction of NS-1-stimulated genes was quantified against the mock control results. The mean value of fold induction (infection/mock, *n* = 3 each) of each gene was visualized and colored.

DENV NS1 is a virotoxin that directly induces platelet apoptosis (23). Secreted NS1 stimulates peripheral blood mononuclear cells to induce the expression of TNF-α, IL-1β, and IL-6 cytokines, which may disrupt the tight junctions of endothelial cells and cause vascular leakage (8). In this study, we measured secreted NS1 in the supernatant of WS-1 cells with DENV-1 or DENV-2 infection, and the pattern of the increasing amount of secreted NS1 was observed in a DENV inoculation dose- and time-dependent manner (Fig. 5A and B). In addition, the secreted NS1 was also expressed in DENV-infected C6/36 cells and HaCaT cells (Fig. S3A and B). We investigated whether DENV NS1 triggers a cell death-associated inflammatory response in dermal fibroblasts. Comparison of cytokine expression levels at 48 h and 72 h in DENV-1 NS1-stimulated WS1 cells revealed a higher level of RNA expression of *IL-18* at 48 h, whereas that for *IL-1β*, *IL-6*, *IL-8*, *TNF-a*, *CXCL10*, *IFN-β*, and *IFIT3* were higher at 72 h (Fig. 5C). In DENV-2 NS1-stimulated WS1 cells, with the exception of *IL-6* and *IL-18*, higher levels of RNA expression were detected for *IL-1β*, *IL-8*, *TNF-a*, *CXCL10*, *IFN-β*, and *IFIT3* at 48 h than at 72 h (Fig. 5D). Interestingly, these results pertaining to the activity of DENV-1 NS1 and DENV-2 NS1 showed that a distinct pattern could be noted for the levels of cytokines expressed at different time points and were similar to the results of the analysis of inflammation caused by DENV-1 and DENV-2 virion infection in WS1 cells (Fig. 2A and B), in which DENV-1 induced intense inflammation in the late phase and DENV-2 induced inflammation at the early phase and was downregulated at the late phase.

### DENV NS1 induced pyroptosis activation in dermal fibroblasts.

**FIG 6:**
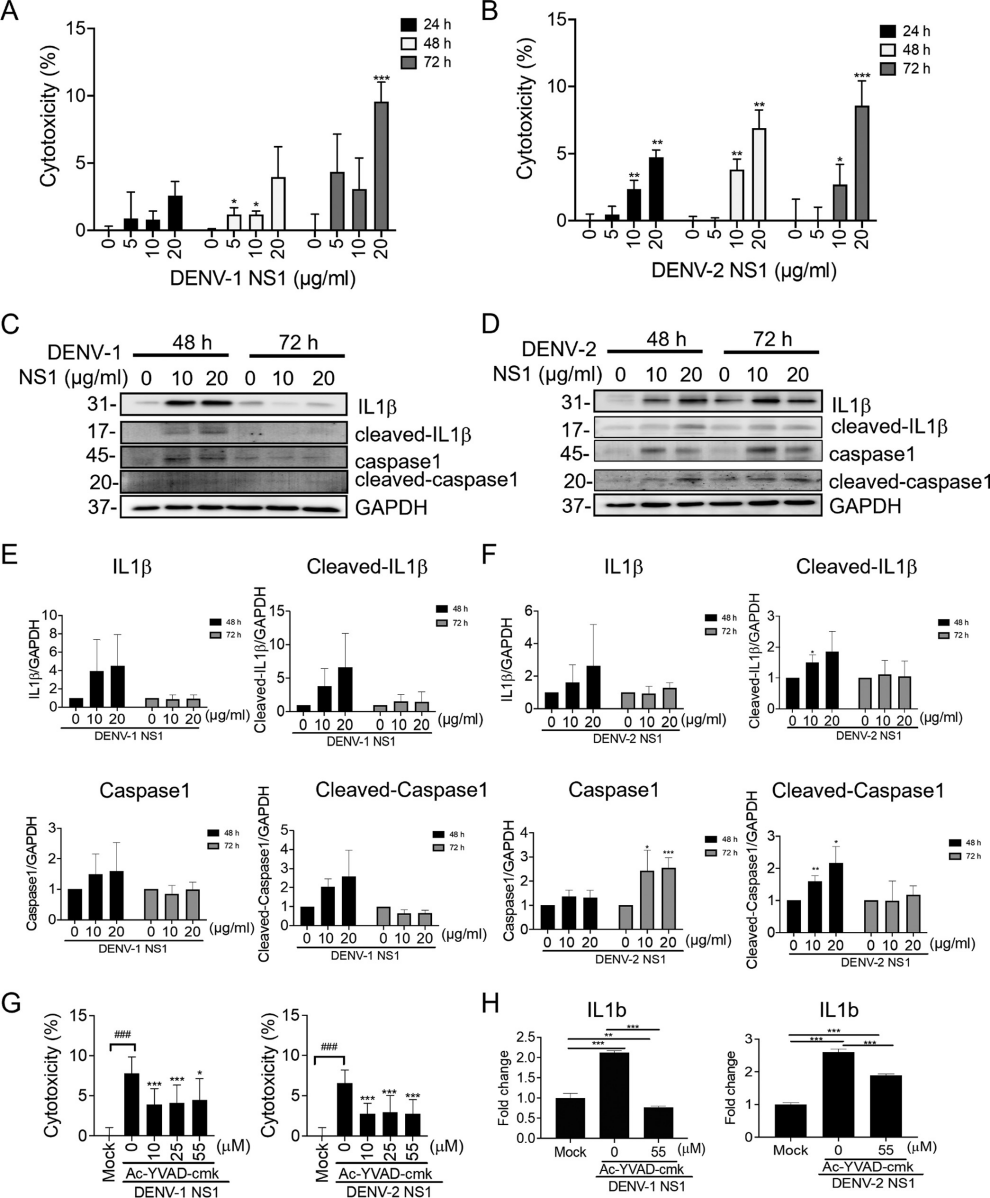
DENV NS1 induced pyroptosis activation in dermal fibroblasts. (A and B) WS1 cells were treated with DENV-1 NS1 or DENV-2 NS1 recombinant proteins (5 to 20 μg/mL) or medium control for 24, 48, and 72 h and then subjected to an LDH release assay. The cytotoxicity was calculated with comparison to an unstimulated control. Data are represented as means ± SD from three independent experiments. Student's *t* test was used for analysis. *, *P* < 0.05; **, *P* < 0.01; ***, *P* < 0.005 (compared with medium control group at each time point). (B and C) Immunoblotting was conducted using the lysates harvested from WS1 after NS-1 treatment for 24 and 48 h. The protein expression levels of caspase-1, cleaved caspase-1, IL-1β, cleaved-IL-1β, and GAPDH were determined. (E and F) Quantification of three independent experiments of immunoblotting, shown as means ± SD. *, *P* < 0.05; **, *P* < 0.01; ***, *P* < 0.01. (G) WS-1 cells were pretreated with Ac-YVAD-cmk (10, 25, and 55 μM) for 1 h and then stimulated with 20 μg/mL DENV-1 NS1 or DENV-2 NS1 for 24 h. The cytotoxicity assay (LDH assay) was conducted. The results were normalized with those for the unstimulated control group. (H) Ac-YVAD-cmk-pretreated WS1 cells were stimulated with recombinant DENV-1 or DENV-2 NS1 (20 μg/mL) for 48 h. The mRNA expression level of *IL1b* was determined using RT-PCR. The results were normalized with the internal control gene, *GAPDH*. Quantification of three independent experiments of immunoblotting is shown as means ± SD. *, *P* < 0.05; **, *P* < 0.01; ***, *P* < 0.01.

To determine whether DENV NS1 plays a role in triggering pyroptosis in dermal fibroblasts, DENV-1 and DENV-2 NS-1-induced cell damage was analyzed in an LDH assay, which showed that DENV-1 and DENV-2 NS-1 induced cytotoxicity in a time- and dose-dependent manner in WS1 cells (Fig. 6A and B). We further evaluated whether NS1 activates caspase-1 and IL-1β. Immunoblotting and quantitation analyses showed that DENV-1 NS-1 transiently induced the protein expression of full-length caspase-1 and IL-1β as well as that of cleaved caspase-1 and IL-1β at 48 h, which then decreased at 72 h (Fig. 6C and D). Similarly, DENV-2 NS1 also enhanced the protein levels of caspase-1 and IL-1β and their active forms, cleaved caspase-1 and IL-1β, at 48 h poststimulation. Except for caspase 1, the expression levels of IL-1β and cleaved caspase-1 and IL-1β similarly decreased at 72 h after stimulation (Fig. 6E and F). The DENV NS1-mediated cytotoxicity and IL-1b mRNA expression were downregulated by the caspase-1 inhibitor Ac-YVAD-cmk (Fig. 6G and H). These results revealed the potential role of NS1 in DENV infection-induced cell death-associated inflammation in dermal cells.

## DISCUSSION

Dermal fibroblasts are the primary cell type to get infected with DENV from mosquito bites. A previous study showed that DENV-2 causes dengue of higher severity than does DENV-1 in humans. To understand the possible difference in mechanisms between the modes of DENV-1 and DENV-2 infections in dermal cells, the cellular responses of dermal fibroblasts infected with DENV-1 and DENV-2 were investigated. We found that dermal fibroblast WS1 cells were susceptible to DENV-1 and DENV-2. However, DENV-2 caused more severe cell damage than did DENV-1, which affected the reduction in virion production in WS1 cells. We detected a strong inflammatory innate immune response and cell death induced by DENV-2 during the early infection period; however, the peak of inflammatory response induced by DENV-1 was detected during the late infection period. Our data showed that DENV-1 activated a higher level of pyroptosis signaling than did DENV-2. In addition, caspase-1 inhibition reduced early DENV replication in the dermal fibroblasts. Thus, in the early infection period, caspase-1 signaling plays a critical role in viral spread. We further revealed that DENV-1 and DENV-2 NS1 protein promoted cell death, inflammatory responses, and the activation of pyroptosis in dermal fibroblasts. In conclusion, these data suggest that NS1 activates cellular pyroptosis to facilitate DENV replication in dermal fibroblasts.

Here, we demonstrated that DENV infection of the skin fibroblasts modulates the inflammatory cytokines as well as cell death; these results are consistent with our previous study (24). Moreover, a different pattern of cellular response was detected in WS1 cells between DENV-1 and DENV-2 infections at the same viral load, in that DENV-2 elicited a stronger inflammatory response than did DENV-1. Commonly, viruses require infected cells for successful replication in order to subsequently infect the neighboring cells, and early during DENV-1 infection, delayed cell death plays an essential role in halting viral replication. In DENV-2 infection, cell death serves as part of the host defense system to limit the replication of the virus within an already-infected host cell (25). However, some viruses have found ways to enhance their replication by exploiting or hijacking specific types of cell death pathways (6). Determining if a certain type of cell death would be friendly or hostile in nature to the patient in the wake of a DENV infection of the fibroblasts is an intriguing topic that requires more study.

Normally, inflammation is a cellular response to viral infection; however, excessive inflammation can lead to tissue damage and exacerbate disease. Strong DENV-mediated systemic inflammation is associated with dengue disease (26), and the nature of the disease is more severe when caused by DENV-2 than when caused by DENV-1 (4, 5). Here, we investigated this phenomenon in dermal fibroblasts with the aid of quantitative real-time PCR (qRT-PCR) to analyze cellular inflammation and antiviral responses and found that the top four genes induced were *IL-8*, *IL-6*, *CXCL-10*, and *IFIT3*, followed by *IFN-β*, *IRF1*, *IFN-α* or *TNF-α*, *viperin*, and *IL-1β* in dermal fibroblasts due to DENV-1 and DENV-2 infection. The activation of two critical transcription factors, IRF3 and NF-κB p65, was detected in infected dermal fibroblasts as well as in other cell types (27). Importantly, DENV-2 induced high levels of expression of these transcripts in 24 to 72 h of infection; however, in the case of DENV-1, excess levels of these transcripts were observed after 72 h of infection. These data are partly supported by an infection model in immunocompetent wild-type mice, which show a stronger IFN-associated innate immune response to DENV-2 infection but weaker response to DENV-1 infection at the early infection phase (28). Our data might reflect the pathogenic differences between DENV-1 and DENV-2 infections. Because the first round or second round of early DENV-2 replication in cells was not defined in this study, we divided the early phase or late phase of infection at the time point of 24 h postinfection instead. Our data implied that the inflammasome-mediated cell death may be essential for releasing a maximal virus into the supernatant and thus higher infectious virus was detected in subsequent rounds of infection; a more precise investigation of this should be conducted in the future.

In generally, apoptosis and autophagy do not cause inflammation; however, pyroptosis is a lytic process that is accompanied by inflammation (7, 12, 29). Apoptosis and pyroptosis have been reported to occur during DENV replication in different cell types, such as hepatocytes, neuronal cells, endothelial cells, and monocytes (30). However, it has never been studied in skin cells, such as keratinocytes and dermal fibroblasts. As DENV-infected dermal fibroblasts undergo cell death with inflammation, pyroptosis may play an important role in this process, as observed in this study. We found that a caspase-1 inhibitor blocked pyroptosis activation and suppressed DENV virion production, suggesting that pyroptosis may be an essential cellular activity for DENV replication. A similar observation has been reported in hepatitis C virus (HCV) infection and showed that pyroptosis contributed to viral release from cells in HCV infection (31). Thus, pyroptosis plays a role that is beneficial to the virus and contributes to the pathogenesis of chronic HCV infection (31). In another case, a caspase-1 inhibitor alleviated the disease conditions of virus-infected mice by significantly decreasing the replication of enterovirus 71 and coxsackievirus B3 and downregulating the expression of caspase-1 (32). Together, these results suggest that pyroptosis is involved in the pathogenesis of viral infections and that treatment with a caspase-1 inhibitor is beneficial to the host response against viral infection.

DENV NS1 is known to be associated with the pathogenesis of severe dengue and induces the vascular endothelium and immune cells to release vasoactive cytokines that cause endothelial hyperpermeability and vascular leakage (33). In this study, we revealed that in addition to the previous known functions of DENV NS1 protein, it also acts as a pyroptosis inducer in dermal fibroblasts. Besides NS1, other DENV viral proteins, such as the envelope protein domain III, induce pyroptosis and inflammasome activation in neutrophils and platelets, which leads to NETosis-mediated inflammation, platelet cell death, and thrombocytopenia (34, 35). In our study, we could not exclude the NS1 present in our viral stock produced from C6/36 cells, but the increased level of the secreted form of NS1 was detected in the WS-1 cells with DENV infection in a dose- and time-dependent manner that would support our finding. DENV-2 NS2A and NS2B mediated inflammasome activation in dermal microvascular endothelial cells (HMEC-1). These studies suggested that multiple DENV viral proteins can trigger the pyroptosis pathway in infected cells.

In conclusion, we demonstrated that the viruses DENV-1 and DNEV-2 and their viral protein NS1 activate different patterns of the cellular pyroptosis response to regulate virus replication in dermal fibroblasts. Hence, we conclude that this response at the DENV primary inoculation site plays a role in the establishment of DENV early infection. Moreover, the discrepancy in the level of pyroptosis activation at different time points between DENV-1 and DENV-2 may be correlated with disease severity.

## MATERIALS AND METHODS

### Cell line, recombinant proteins, and chemicals.

WS1 cells were established from normal Homo sapiens skin fibroblast cells (BCRC 60300, Bioresource Collection and Research Center of Hsinchu, Taiwan) and were grown in minimum essential medium (MEM) containing 10% heat-inactivated fetal bovine serum (FBS; Gibco, Thermo Fisher Scientific, Waltham, MA, USA) at 37°C in 5% CO_2_. C6/36 mosquito cells (CRL-1660; ATCC, Manassas, VA, USA) were grown in RPMI 1640 medium with 5% FBS at 28°C in 5% CO_2_. BHK21 cells (baby hamster kidney fibroblasts; BCRC 60041) were grown in Dulbecco's modified Eagle medium with 5% FBS at 37°C in 5% CO_2_. DENV-1 NS1 recombinant protein derived from strain Nauru/Western Pacific/1974 (ENZ-PRT104-0100) and DENV-2 NS1 recombinant protein derived from strain Thailand/16681/84, (ENZ-PRT105-0100) were from Enzo Life Sciences Inc. (Farmingdale, NY, USA). This study used Ac-YVAD-cmk (caspase-1 inhibitor inh-yvad, San Diego, CA, USA) and imipramine hydrochloride (catalog number I7379, Sigma-Aldrich).

### DENV infection and inhibitors treatment.

DENV-1 766733A and DENV-2 PL046 (GenBank accession number AJ968413.1) were isolated from patients with dengue fever in Taiwan (36). DENV was propagated in C6/36 mosquito cells, and the virus titer was quantified in BHK21 cells. Before viral infection, WS1 cells (5 × 10^4^ cells/well) were grown in 12-well plates and incubated for 16 h. The cells were inoculated with DENV-1 or DENV-2 at different MOIs of 0.1, 5, and 10. After viral adsorption for 2 h, the cell medium was replaced with complete growth medium. The analyses of infection were performed at 24, 48, and 72 h postinfection. In some cases, caspase-1 inhibitor Ac-YVAD-cmk (55 μM) and imipramine anticholinergic (10, 25, 50, 75, or 100 μM) were added into the WS-1 cells for 24 h before DENV infection.

### Immunofluorescence assay.

Mock- or DENV-infected WS1 cells were fixed with 4% paraformaldehyde for 10 min, followed by cell membrane permeabilization with 0.5% Triton X-100 for 10 min. After rinsing with phosphate-buffered saline (PBS), the cells were treated with 10% skim milk in PBS for 30 min. Cells were incubated with anti-DENV NS3 (1:500; catalog number YH0034, Yao-Hong Biotechnology, Taipei, Taiwan), anti-NF-κB p65 (C-20; 1:500; catalog number sc-372, Santa Cruz Biotechnology, Santa Cruz, CA, USA) or anti-IRF3 (FL-425; catalog number sc-9082, Santa Cruz Biotechnology) individually, at 4°C overnight. After three PBS washes, the cells were stained with goat anti-mouse IgG Alexa Fluor 488-conjugated secondary antibody (catalog number A11001, Invitrogen, ThermoFisher Scientific, Waltham, MA, USA) or Alexa Fluor 568-conjugated goat anti-rabbit IgG (catalog number A-11036, Invitrogen, ThermoFisher) for 2 h. Nuclear DNA was stained with 4′,6-diamidino-2-phenylindole (DAPI) for 5 min. The fluorescence signals were captured and quantified by fluorescence microscopy (Axio Observer A1, Zeiss, Oberkochen, Germany).

### Detection of cell viability.

A CellTilter-Glo luminescent cell viability assay (catalog number G7571, Promega, Madison, WI, USA) was used to measure cell proliferation with ATP synthesis. WS1 cells (1 × 10^4^/well) in 96-well plate were incubated overnight. WS1 cell culture medium was replaced with serum-free medium followed by the infection with DENV-1 or DENV-2 at MOIs 0.1, 1, 5, and 10 or mock infected. After 2 h adsorption, the virus supernatants were removed and cells were maintained with MEM culture medium. After 24, 48, or 72 h of incubation, CellTiter-Glo reagent was added into the cell culture medium, and the luminescence was measured in a TriStar LB 941 multimode microplate reader from Berthold Technologies (Bad Wildbad, Germany). The intensity of the luminescence signals (in relative light units [RLU]) indicated the cell viability or proliferation. All experiments were performed in triplicate wells for each condition and repeated at least three times.

### LDH cytotoxicity assay.

The LDH cytotoxicity assay (catalog number ab65393, Abcam, Cambridge, MA, USA) was used to detect LDH released from damaged cells. WS1 cells (1 × 10^4^/well) were pretreated with vehicle or incremental concentrations (0, 5, 10, 25, 55, and 100 μM) of Ac-YVAD-cmk for 24 h before exposure to DENV-2 at an MOI of 1. After 2 h of adsorption, cell medium was replaced with 10% FBS in MEM containing drugs. After 24 h and 48 h, the LDH cytotoxicity assay was performed. WS1 cells (1 × 10^4^/well) were stimulated with different amounts of DENV-1 NS1 and DENV-2 NS1 (0, 2.5, 5, 10, or 20 μg/mL) for 24 h, 48 h, and 72 h. The cell culture supernatants were mixed with LDH substrate and incubated for 30 min at room temperature. The absorbance of formazan products was measured at 450 nm by using an EPOCHTM 2 microplate reader (BioTek, Winooski, VT, USA). Experiments were performed in triplicate and repeated three times.

### RNA isolation and qRT-PCR analysis.

Total RNA was extracted from DENV-infected cells or mock-infected cells by adding TRIzol reagent (catalog number 15596026, Invitrogen, ThermoFisher Scientific) according to the manufacturer’s protocol. Five micrograms of total RNA of each sample was used to synthesize cDNA with a SuperScript III reverse transcriptase kit (catalog number 18080093, Invitrogen, ThermoFisher Scientific). Real-time PCR was performed with 5 ng cDNA, 3 μM specific primers, and SYBR green PCR master mix (catalog number 4312704, Applied Biosystems, ThermoFisher Scientific) in a final reaction volume of 10 μL. The RT PCR amplification program involved activation at 95°C for 20 min followed by 40 amplification cycles of 95°C for 3 s and 60°C for 1 s. Data were analyzed by using StepOnePlus software from Applied Biosystems. Gene expression levels (fold change) were normalized to that of the endogenous reference gene glyceraldehyde 3-phosphate dehydrogenase (GAPDH) and were calculated as 2^−△△^*^CT^* (△△*C_T_* = △*C_T_* of sample − △*C_T_* of GAPDH). The primer pair sequences are shown in Table S1.

### In-cell Western assay.

The In-cell Western assay is a quantitative immunofluorescence assay performed in multiwell plates. WS1 cells were pretreated with vehicle or imipramine at incremental concentrations (10, 25, 50, 75, or 100 μM) for 24 h before exposure to DENV-1 or DENV-2 at an MOI of 1. After 2 h of adsorption, cells were cultured with 10% FBS in MEM containing drugs for 24 h. Mock control or infected cells were fixed with 4% paraformaldehyde for 5 min and then permeabilized with 0.5% Triton X-100 for 10 min at room temperature. After blocking buffer treatment, the cells were incubated with a mouse DENV-NS3 antibody (catalog number YH0034, Yao-Hong Biotechnology, Taiwan) for 4°C overnight. After triple washes with PBS, the cells were stained with a 1:1,000 dilution of a donkey anti-mouse IgG IRDye 800cw antibody (catalog number 926-32212, LI-COR Biosciences, Lincoln, NE, USA) at room temperature for 2 h. Microplates were protected from light at 4°C. Microplates were scanned with an Odyssey CLx imaging system (LI-COR Biosciences) to detect the level of fluorescent intensity of DENV-NS3. The integrated fluorescence intensity represented the protein expression levels. The IC_50_ from dose-response curves indicated the virus inhibition. Nonlinear regression analysis with GraphPad Prism software was used to calculate IC_50_ values by plotting log inhibitor versus normalized response (variable slope).

### Immunoblotting assay.

WS1 cells were lysed with protein lysis buffer (2% SDS, 50 mM Tris-HCl [pH 7.5]) containing protease inhibitor and phosphatase inhibitor cocktail (Roche, Basel, Switzerland). Eighty micrograms of protein lysate was separated with 10% acrylamide gel in SDS-PAGE and transferred to a polyvinylidene difluoride (PVDF) membrane. PVDF membranes were blocked with 5% milk in Tris-buffered saline with Tween 20 (TBST) for 1 h at room temperature and then incubated with primary antibody overnight at 4°C. After TBST washes, the membranes were incubated with horseradish peroxidase-conjugated secondary antibody for 2 h at room temperature. Enhanced chemiluminescent reagent (Advansta, San Jose, CA, USA) was used for blot detection. Image and emission signal were captured and quantified by a BioSpectrum image system (UVP, Upland, CA, USA). The primary antibodies were anti-DENV-NS3 (catalog number GTX124252, GeneTex, Hsinchu, Taiwan), anti-phospho-IRF3 (catalog number 2562-1, EPITOMICS), or anti-phospho-NF-κB p65 (Ser536; catalog number 3033, Cell Signaling), anti-NF-κB p65 (C-20; catalog number sc-372, Santa Cruz Biotechnology, CA, USA), anti-NLRP3 (catalog number 15101, Cell Signaling), anti-pro-caspase-1 (catalog number ARG57293, Arigo Biolaboratories), anti-pro-IL-1β (catalog number GTX130021, GeneTex), anti-cleaved IL-1β (catalog number 83186, Cell Signaling), and anti-GAPDH (catalog number 60004-1-Ig, Proteintech, Rosemont, IL, USA).

### Plaque assay and focus-forming assay.

To determine virus titers, culture medium from DENV-infected WS-1 cells was harvested for plaque assay. DENV dilutions were added to 80% confluent BHK21 cells and incubated at 37°C for 2 h. After adsorption, cells were washed and overlaid with 1% agarose containing culture medium with 2% FBS. After 7 days of incubation, cells were fixed with 10% formaldehyde and stained with 0.5% crystal violet. The focus-forming assay (FFA) is an immunostaining technique and a variation of the viral plaque assay. In some cases, we performed FFA to determine DENV titers. The agarose-overlaid BHK-21 cells with DENV infections were harvested at 72 h postinoculation for immunofluorescence assay with the mouse DENV-NS3 antibody (catalog number YH0034, Yao-Hong Biotechnology) followed by anti-mouse IgG IRDye 800cw antibody (catalog number 926-32212, LI-COR Biosciences). The plates were scanned with an Odyssey CLx imaging system (LI-COR Biosciences) to detect the DENV focuses.

### ELISA for dengue virus NS1.

For the enzyme-linked immunosorbent assay (ELISA), WS1 cells were infected with DENV-1 or DENV-2 at an MOI of 0.1, 1, 5, and 10. After viral adsorption for 2 h, the media were replaced with MEM culture medium with 10% FBS and incubated at 37°C in 5% CO_2_. The culture supernatants were harvested at 24 h, 48 h, and 72 h postinfection, and the supernatants were diluted (1:50) for the detection of DENV NS-1 using a dengue virus NS1 ELISA kit (catalog number ARG81357, Arigo Biolaboratories, Hsinchu City, Taiwan). The absorbance of formazan products of the NS1 protein standards, control group, and samples were measured at 450 nm using an EPOCHTM 2 microplate reader (BioTek). The absorbance values were input to GainData software provided by Arigo (4 Parameter Logistics curve fit) to determine the amount of NS1. Experiments were performed in triplicate and repeated three times, and statistical significance was calculated.

### Statistical analysis.

GraphPad Prism software was used for statistical analysis in this study. All data are presented as means ± standard deviations (SD) at least three independent experiments. The significant differences between groups were analyzed by Student's *t* test with GraphPad Prism software (La Jolla, CA, USA). A *P* value of <0.05 was accepted and represented statistical significance. Nonlinear regression analysis was used to calculate IC_50_ values by plotting the log inhibitor concentration versus normalized response.

### Data availability.

The data presented in this study are contained within the article.

## References

[1] Guzman MG, Gubler DJ, Izquierdo A, Martinez E, Halstead SB. 2016. Dengue infection. Nat Rev Dis Primers 2:16055. doi:10.1038/nrdp.2016.55. 27534439

[2] World Health Organization. 2022. Dengue and severe dengue. https://www.who.int/news-room/fact-sheets/detail/dengue-and-severe-dengue. Accessed 7 September 2022.

[3] Centers for Disease Control and Prevention, National Center for Emerging and Zoonotic Infectious Diseases, Division of Vector-Borne Diseases. 2022. Dengue. https://www.cdc.gov/dengue/about/index.html. Accessed 7 September 2022.

[4] Soo KM, Khalid B, Ching SM, Chee HY. 2016. Meta-analysis of dengue severity during infection by different dengue virus serotypes in primary and secondary infections. PLoS One 11:e0154760. doi:10.1371/journal.pone.0154760. 27213782 PMC4877104

[5] Sangkaew S, Ming D, Boonyasiri A, Honeyford K, Kalayanarooj S, Yacoub S, Dorigatti I, Holmes A. 2021. Risk predictors of progression to severe disease during the febrile phase of dengue: a systematic review and meta-analysis. Lancet Infect Dis 21:1014–1026. doi:10.1016/S1473-3099(20)30601-0. 33640077 PMC8240557

[6] Imre G. 2020. Cell death signalling in virus infection. Cell Signal 76:109772. doi:10.1016/j.cellsig.2020.109772. 32931899 PMC7486881

[7] Kroemer G, Galluzzi L, Vandenabeele P, Abrams J, Alnemri ES, Baehrecke EH, Blagosklonny MV, El-Deiry WS, Golstein P, Green DR, Hengartner M, Knight RA, Kumar S, Lipton SA, Malorni W, Nunez G, Peter ME, Tschopp J, Yuan J, Piacentini M, Zhivotovsky B, Melino G, Nomenclature Committee on Cell Death. 2009. Classification of cell death: recommendations of the Nomenclature Committee on Cell Death 2009. Cell Death Differ 16:3–11. doi:10.1038/cdd.2008.150. 18846107 PMC2744427

[8] Suwanmanee S, Luplertlop N. 2017. Immunopathogenesis of dengue virus-induced redundant cell death: apoptosis and pyroptosis. Viral Immunol 30:13–19. doi:10.1089/vim.2016.0092. 27860556

[9] Pan Y, Cheng A, Wang M, Yin Z, Jia R. 2021. The dual regulation of apoptosis by flavivirus. Front Microbiol 12:654494. doi:10.3389/fmicb.2021.654494. 33841381 PMC8024479

[10] El-Bacha T, Midlej V, Pereira da Silva AP, Silva da Costa L, Benchimol M, Galina A, Da Poian AT. 2007. Mitochondrial and bioenergetic dysfunction in human hepatic cells infected with dengue 2 virus. Biochim Biophys Acta 1772:1158–1166. doi:10.1016/j.bbadis.2007.08.003. 17964123

[11] Thepparit C, Khakpoor A, Khongwichit S, Wikan N, Fongsaran C, Chingsuwanrote P, Panraksa P, Smith DR. 2013. Dengue 2 infection of HepG2 liver cells results in endoplasmic reticulum stress and induction of multiple pathways of cell death. BMC Res Notes 6:372. doi:10.1186/1756-0500-6-372. 24034452 PMC3847886

[12] Bergsbaken T, Fink SL, Cookson BT. 2009. Pyroptosis: host cell death and inflammation. Nat Rev Microbiol 7:99–109. doi:10.1038/nrmicro2070. 19148178 PMC2910423

[13] Wu MF, Chen ST, Yang AH, Lin WW, Lin YL, Chen NJ, Tsai IS, Li L, Hsieh SL. 2013. CLEC5A is critical for dengue virus-induced inflammasome activation in human macrophages. Blood 121:95–106. doi:10.1182/blood-2012-05-430090. 23152543

[14] Tan TY, Chu JJH. 2013. Dengue virus-infected human monocytes trigger late activation of caspase-1, which mediates pro-inflammatory IL-1beta secretion and pyroptosis. J Gen Virol 94:2215–2220. doi:10.1099/vir.0.055277-0. 23884363

[15] Rathore APS, St John AL. 2018. Immune responses to dengue virus in the skin. Open Biol 8. doi:10.1098/rsob.180087. PMC6119867 30135238

[16] Bustos-Arriaga J, Garcia-Machorro J, Leon-Juarez M, Garcia-Cordero J, Santos-Argumedo L, Flores-Romo L, Mendez-Cruz AR, Juarez-Delgado FJ, Cedillo-Barron L. 2011. Activation of the innate immune response against DENV in normal non-transformed human fibroblasts. PLoS Negl Trop Dis 5:e1420. doi:10.1371/journal.pntd.0001420. 22206025 PMC3243703

[17] Surasombatpattana P, Hamel R, Patramool S, Luplertlop N, Thomas F, Despres P, Briant L, Yssel H, Misse D. 2011. Dengue virus replication in infected human keratinocytes leads to activation of antiviral innate immune responses. Infect Genet Evol 11:1664–1673. doi:10.1016/j.meegid.2011.06.009. 21722754

[18] Duangkhae P, Erdos G, Ryman KD, Watkins SC, Falo LD, Jr, Marques ETA, Jr, Barratt-Boyes SM. 2018. Interplay between keratinocytes and myeloid cells drives dengue virus spread in human skin. J Invest Dermatol 138:618–626. doi:10.1016/j.jid.2017.10.018. 29106931

[19] Montes-Gomez AE, Garcia-Cordero J, Marcial-Juarez E, Shrivastava G, Visoso-Carvajal G, Juarez-Delgado FJ, Flores-Romo L, Sanchez-Torres MC, Santos-rgumedo L, Bustos-Arriaga J, Cedillo-Barron L. 2020. Crosstalk between dermal fibroblasts and dendritic cells during dengue virus infection. Front Immunol 11:538240. doi:10.3389/fimmu.2020.538240. 33193307 PMC7645109

[20] Chen HR, Lai YC, Yeh TM. 2018. Dengue virus non-structural protein 1: a pathogenic factor, therapeutic target, and vaccine candidate. J Biomed Sci 25:58. doi:10.1186/s12929-018-0462-0. 30037331 PMC6057007

[21] Puerta-Guardo H, Glasner DR, Espinosa DA, Biering SB, Patana M, Ratnasiri K, Wang C, Beatty PR, Harris E. 2019. Flavivirus NS1 triggers tissue-specific vascular endothelial dysfunction reflecting disease tropism. Cell Rep 26:1598–1613.e8. doi:10.1016/j.celrep.2019.01.036. 30726741 PMC6934102

[22] Wichit S, Hamel R, Bernard E, Talignani L, Diop F, Ferraris P, Liegeois F, Ekchariyawat P, Luplertlop N, Surasombatpattana P, Thomas F, Merits A, Choumet V, Roques P, Yssel H, Briant L, Misse D. 2017. Imipramine inhibits Chikungunya virus replication in human skin fibroblasts through interference with intracellular cholesterol trafficking. Sci Rep 7:3145. doi:10.1038/s41598-017-03316-5. 28600536 PMC5466638

[23] Chao CH, Wu WC, Lai YC, Tsai PJ, Perng GC, Lin YS, Yeh TM. 2019. Dengue virus nonstructural protein 1 activates platelets via Toll-like receptor 4, leading to thrombocytopenia and hemorrhage. PLoS Pathog 15:e1007625. doi:10.1371/journal.ppat.1007625. 31009511 PMC6497319

[24] Wei KC, Wei WJ, Liu YS, Yen LC, Chang TH. 2020. Assessment of prolonged dengue virus infection in dermal fibroblasts and hair-follicle dermal papilla cells. Viruses 12:267. doi:10.3390/v12030267. 32121148 PMC7150742

[25] Jorgensen I, Rayamajhi M, Miao EA. 2017. Programmed cell death as a defence against infection. Nat Rev Immunol 17:151–164. doi:10.1038/nri.2016.147. 28138137 PMC5328506

[26] Martina BE, Koraka P, Osterhaus AD. 2009. Dengue virus pathogenesis: an integrated view. Clin Microbiol Rev 22:564–581. doi:10.1128/CMR.00035-09. 19822889 PMC2772360

[27] Chang TH, Liao CL, Lin YL. 2006. Flavivirus induces interferon-beta gene expression through a pathway involving RIG-I-dependent IRF-3 and PI3K-dependent NF-kappaB activation. Microbes Infect 8:157–171. doi:10.1016/j.micinf.2005.06.014. 16182584

[28] Rathore APS, Mantri CK, Tan MW, Shirazi R, Nishida A, Aman SAB, Morrison J, St John AL. 2021. Immunological and pathological landscape of dengue serotypes 1–4 infections in immune-competent mice. Front Immunol 12:681950. doi:10.3389/fimmu.2021.681950. 34168651 PMC8219075

[29] Kofahi HM, Taylor NG, Hirasawa K, Grant MD, Russell RS. 2016. Hepatitis C virus infection of cultured human hepatoma cells causes apoptosis and pyroptosis in both infected and bystander cells. Sci Rep 6:37433. doi:10.1038/srep37433. 27974850 PMC5156923

[30] Begum F, Das S, Mukherjee D, Mal S, Ray U. 2019. Insight into the tropism of dengue virus in humans. Viruses 11:1136. doi:10.3390/v11121136. 31835302 PMC6950149

[31] Wallace HL, Wang L, Gardner CL, Corkum CP, Grant MD, Hirasawa K, Russell RS. 2022. Crosstalk between pyroptosis and apoptosis in hepatitis C virus-induced cell death. Front Immunol 13:788138. doi:10.3389/fimmu.2022.788138. 35237259 PMC8882739

[32] Wang Y, Qin Y, Wang T, Chen Y, Lang X, Zheng J, Gao S, Chen S, Zhong X, Mu Y, Wu X, Zhang F, Zhao W, Zhong Z. 2018. Pyroptosis induced by enterovirus 71 and coxsackievirus B3 infection affects viral replication and host response. Sci Rep 8:2887. doi:10.1038/s41598-018-20958-1. 29440739 PMC5811489

[33] Glasner DR, Puerta-Guardo H, Beatty PR, Harris E. 2018. The good, the bad, and the shocking: the multiple roles of dengue virus nonstructural protein 1 in protection and pathogenesis. Annu Rev Virol 5:227–253. doi:10.1146/annurev-virology-101416-041848. 30044715 PMC6311996

[34] Lien TS, Sun DS, Hung SC, Wu WS, Chang HH. 2021. Dengue virus envelope protein domain III induces Nlrp3 inflammasome-dependent NETosis-mediated inflammation in mice. Front Immunol 12:618577. doi:10.3389/fimmu.2021.618577. 33815373 PMC8009969

[35] Lien TS, Chan H, Sun DS, Wu JC, Lin YY, Lin GL, Chang HH. 2021. Exposure of platelets to dengue virus and envelope protein domain III induces Nlrp3 inflammasome-dependent platelet cell death and thrombocytopenia in mice. Front Immunol 12:616394. doi:10.3389/fimmu.2021.616394. 33995345 PMC8118162

[36] Lin YL, Liao CL, Chen LK, Yeh CT, Liu CI, Ma SH, Huang YY, Huang YL, Kao CL, King CC. 1998. Study of dengue virus infection in SCID mice engrafted with human K562 cells. J Virol 72:9729–9737. doi:10.1128/JVI.72.12.9729-9737.1998. 9811707 PMC110483

