# Supplementary material for: Discrepant Activation Pattern of Inflammation and Pyroptosis Induced in Dermal Fibroblasts in Response to Dengue Virus Serotypes 1 and 2 and Nonstructural Protein 1
Source: Microbiol Spectr. 2023 Jan 11;11(1):e03586-22. doi: 10.1128/spectrum.03586-22 (PMC9927091; doi:10.1128/spectrum.03586-22)
Supplement: Supplemental file 1 — Supplemental material. Download spectrum.03586-22-s0001.pdf, PDF file, 0.4 MB [file spectrum.03586-22-s0001.pdf]

Supplementary information

Table S1. The RT-qPCR primers used in this study.

	Primer sequences
IFIT3	F: 5′-GCTGAAGGAGAGCAGTTTGTTGA-3′<br>R: 5′-AGGACATCTGTTTGGCAAGGA-3′
IFNα	F: 5′-CCTCGCCCTTTGCTTTACTG-3′<br>R: 5′-CAGAGAGCAGCTTGACTTGCA-3′
IFNβ	F: 5′-TGAGCAGTCTGCACCTGAAA-3′<br>R: 5′-GCTTGAAGCAATTGTCCCGT-3′
Viperin	F: 5′-CAAGGAAGAATGTGAGCAAGAGTAGA-3′<br>R: 5′-TGATATGGTGACATGGCTTCACT-3′
IL8	F: 5′-ACACTGCGCCAACACAGAAATTA-3′<br>R: 5′-TTTGCTTGAAGTTTCACTGGCATC-3′
IL6	F: 5′-TGTCCTGCAGCCACTGGTTC-3′<br>R: 5′-AAGCCAGAGCTGTGCAGATGAGTA-3′
CXCL10	F: 5′-TGAGCCTACAGCAGAGGAACCT-3′<br>R: 5′-TGAATGCCACTTAGAGTCAGAAAGA-3′
TNFα	F: 5′-GACAAGCCTGTAGCCCATGTTGTA-3′<br>R: 5′-CAGCCTTGGCCCTTGAAGA-3′
IL23p19	F: 5′-ACAACTGAGGGAACCAAACCA-3′<br>R: 5′-CCACTTGCTTTGAGCCTGATT-3′
TRAF3	F: 5′-CTGCTTCCTTGGCCGTTTAA-3′<br>R: 5′-CGAGAGGACCCCTGATCCA-3′
IL1β	F: 5′-ACGATGCACCTGTACGATCACT-3′<br>R: 5′-CACCAAGCTTTTTTGCTGTGAGT
MAD5	F: 5′-TGCTGGACTACCTGACCTTTCTG-3′<br>R: 5′- GGCGACTGTCCTCTGAATCTG-3′
MAVS	F: 5′-TGGATGTTGTAGAGATTCTGCCTT A-3′<br>R: 5′-CGCTGAAGGGTATTGAAGAGATG-3′
HMGB1	F: 5′-ACGCTGACGAAAGAGACCTG-3′<br>R: 5′-GAGAGCGGACTACGGATGC-3′
IRF1	F: 5′-GGCATCCCGCCTGAACT-3′<br>R: 5′-GCGCCTGGCCGAAAG-3′
IRF2	F: 5′-CAGATCTCCCCCGTGTCTTC-3′<br>R: 5′-TCATCGCTGGGCACACTATC-3′
IRF3	F: 5′-TTCCCGGGAGGGATAAGC-3′<br>R: 5′-GGGCAGAGCGGAAATTCC-3′
IRF4	F: 5′-GGCCAACCCTCCTCCAAT-3′

	R: 5′-TTGCCTGTTAAGTCCCATCTGTCT-3′
IRF5	F: 5′-GGGTGGGCTAGCATTGCA-3′<br>R: 5′-ATAGCACAGGAATATACACAAGAGAGTGA-3′
IRF6	F: 5′-CCAGGATCGAGCTCTGTTTGAC-3′<br>R: 5′-CCTCTCACTTCCCCCATCAG-3′
IRF7	F: 5′-TCCCCACGCTATACCATCTACCT-3′<br>R: 5′-ACAGCCAGGGTTCCAGCTT-3′
IRF8	F: 5′-TGGCCTGGATGCTGTAACC-3′<br>R: 5′-GATAATGACGGGTAAAGGATAGAAAATAA-3′
IRF9	F: 5′-GAGTGCGTGGAGCTCTTCAGA-3′<br>R: CCCTGAAAGTACCTGACCAAGTCT-3′
GAPDH	F: 5′-CAACTGGTCGTGGACAACCAT-3′<br>R: 5′-GCACGGACACTCACAATGTTC-3′
IL18	F: 5′- TCTTCATTGACCAAGGAAATCGG-3′<br>R:5′- TCCGGGGTGCATTATCTCTAC-3′

Figure S1

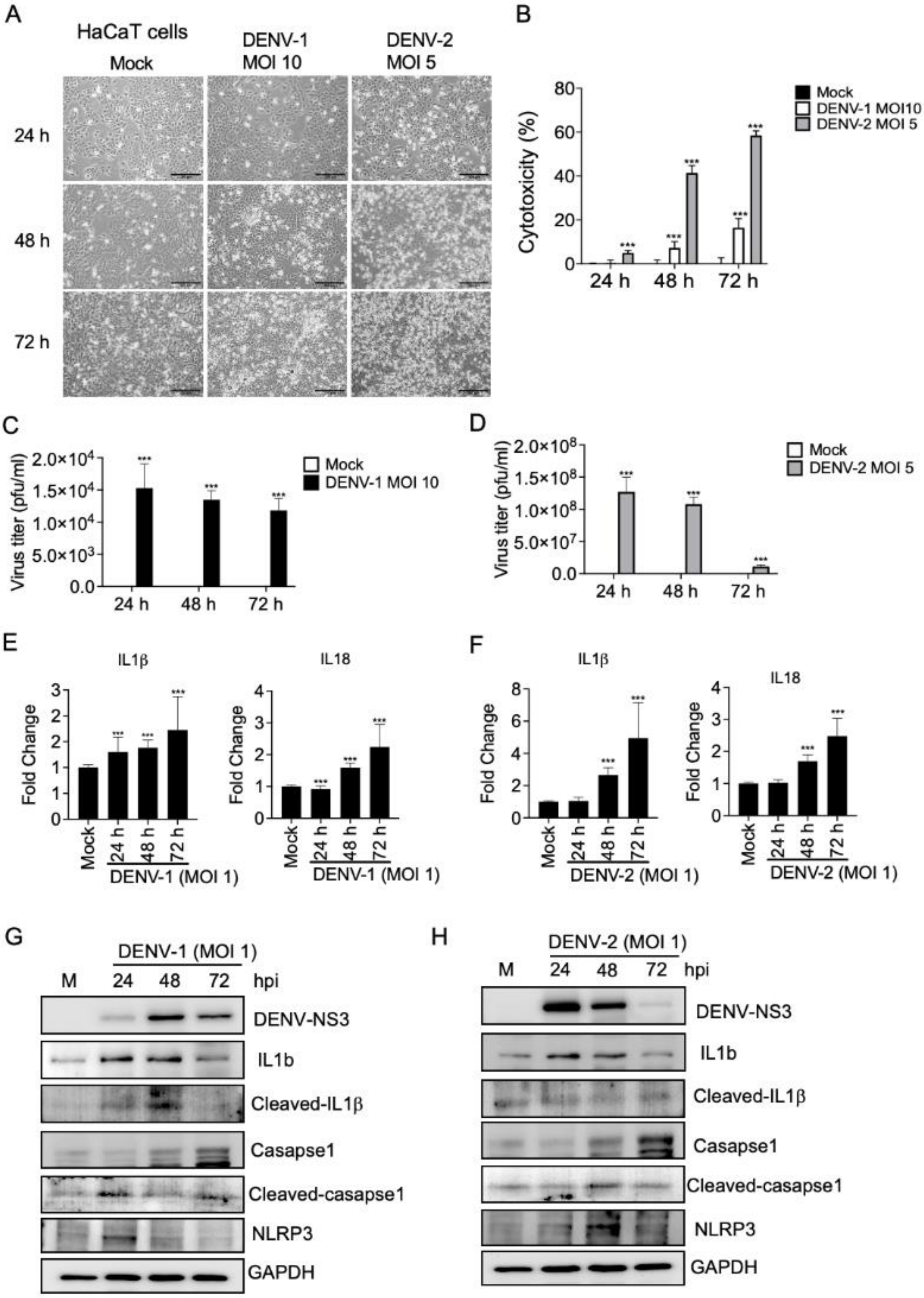
A
HaCaT cells
Mock
DENV-1
MOI 10
DENV-2
MOI 5
24 h
48 h
72 h
B
Cytotoxicity (%)
Mock
DENV-1 MOI10
DENV-2 MOI 5
24 h
48 h
72 h
C
Virus titer (pfu/ml)
Mock
DENV-1 MOI 10
D
Virus titer (pfu/ml)
Mock
DENV-2 MOI 5
E
IL1β
IL18
Fold Change
Mock
DENV-1 (MOI 1)
F
IL1β
IL18
Fold Change
DENV-2 (MOI 1)
G
DENV-1 (MOI 1)
M
24
48
72
hpi
DENV-NS3
IL1b
Cleaved-IL1β
Casapse1
Cleaved-casapse1
NLRP3
GAPDH
H
DENV-2 (MOI 1)
M
24
48
72
hpi
DENV-NS3
IL1b
Cleaved-IL1β
Casapse1
Cleaved-casapse1
NLRP3
GAPDH

Figure S1. DENV-1 and DENV-2 infection in skin keratinocyte, HaCaT cells.

(A) 5 x 10^5 HaCaT cells were un-infected (mock) or infected with DENV-1 (MOI 10) or DENV-2 (MOI 5) for 24 h, 48 h and 72 h. The morphology of HaCaT cells was captured by a phase contrast microscope. 100 x magnification; scale bar = 100 μm. (B) Lactate dehydrogenase (LDH) assays revealed cytotoxicity in DENV-infected cells. (C and D) Virions production by HaCaT cells was tittered by plaque assay. HaCaT cells were infected with DENV-1 or DENV-2 at MOI 1 for 24 h, 48 h and 72 h. The culture medium was harvested for plaque assay. (E and F) IL1β and IL18 gene expression were determined using RT-qPCR. The results were normalized with the internal control gene, GAPDH. The fold induction was quantified against mock control. (G) The protein expression of DENV-NS3, IL1β, cleaved-IL1β, caspase1, cleaved caspase1, NLRP3 and internal control, GAPDH, were identified by immunoblotting assay.

Fugure S2

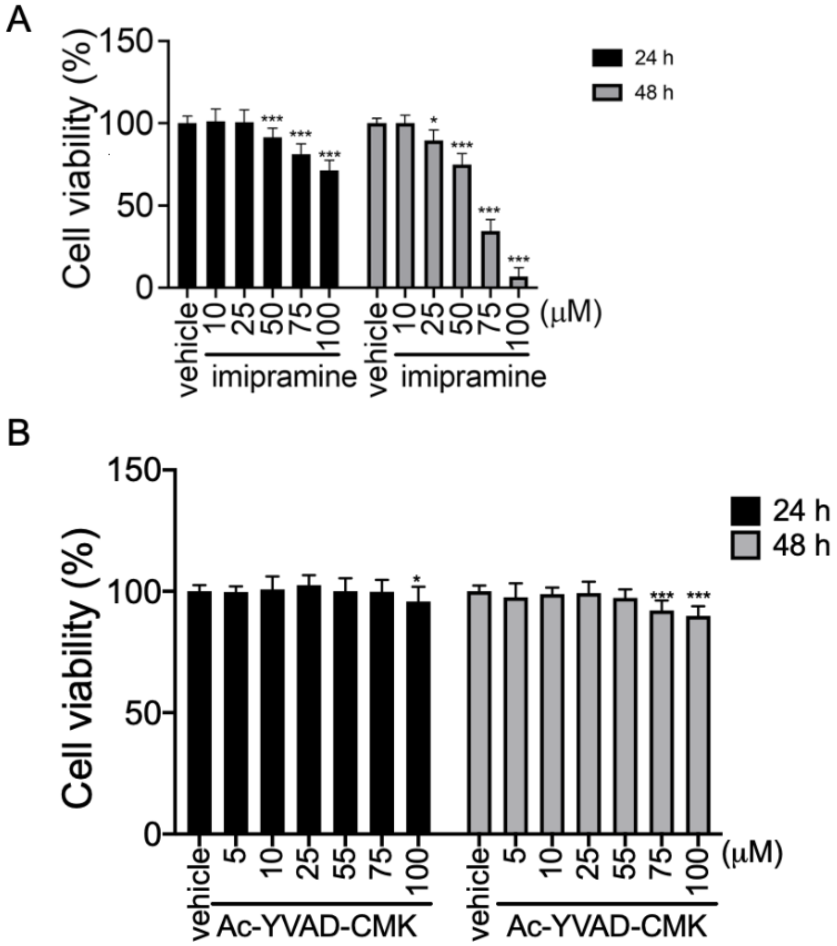

Figure S2. The cell viability of WS1 cells with imipramine and Ac-yvad-cmk treatment.
WS1 cells were treated with vehicle control (DMSO) or the indicated concentrations of imipramine (A) and caspase-1 inhibitor Ac-yvad-cmk (B) for 24 h or 48 h. Cell viability was evaluated by CellTiter-Glo luminescent cell viability assay. The results are presented as mean ± standard deviation of three separate experiments. Statistical Student's t-test, *, $p < 0.05$; **, $p < 0.01$; ***, and $p < 0.005$ compared with mock control.

Figure S3

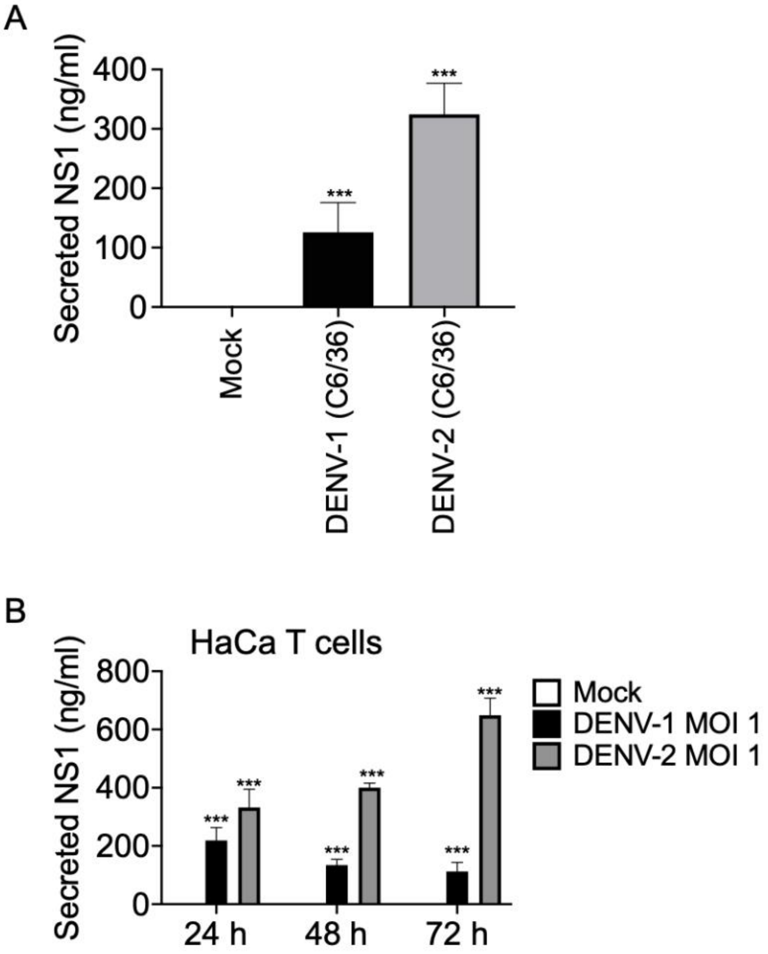

Figure S3. Detection of the secreted NS1 in the culture medium of DENV-infected cells. (A) DENV-1 or DENV-2 virus were propagated in a C6/36 mosquito host cell line; the harvested virus supernatant was subjected to NS1 detection. (B) HaCaT cells were un-infected (mock) or infected with DENV-1 or DENV-2 at MOI of 1 for 24 h, 48 h and 72 h. The culture supernatants from C6/36 and HaCaT cells were diluted with culture medium (1:50) for detection of NS1 concentration by dengue virus NS1 ELISA kit. All data were presented as mean ± standard deviation of three separate experiments. Statistical Student's t-test, *, $p < 0.05$; **, $p < 0.01$; ***, and $p < 0.005$ compared with mock control.
